# The use of bone turnover markers for monitoring the treatment of osteoporosis in postmenopausal females undergoing total knee arthroplasty: a prospective randomized study

**DOI:** 10.1186/s13018-021-02343-3

**Published:** 2021-03-17

**Authors:** Rui Ma, Mengjun Wu, Yongwei Li, Jialin Wang, Pei Yang, Yuanyuan Chen, Wei Wang, Jinhui Song, Kunzheng Wang

**Affiliations:** Department of Bone and Joint Surgery, The Second Affiliated Hospital of Xi’an Jiaotong University, NO. 157 Xiwu Road, Xi’an, 710004 Shaanxi People’s Republic of China

**Keywords:** Bone turnover markers, Postmenopausal osteoporosis, Total knee arthroplasty, Monitoring treatment, Osteoarthritis

## Abstract

**Background:**

Osteoporosis (OP) and osteoarthritis (OA) commonly coexist in postmenopausal females. The decrease in bone density and increase in bone resorption in postmenopausal females with OP may consequently affect the surgical outcome of total knee arthroplasty (TKA). However, clinicians often ignore monitoring the treatment of OP in the perioperative management of TKA. Bone turnover marker (BTM) can timely and accurately reflect bone metabolism to monitor the treatment of OP. The purpose of this study was to investigate the effect of BTM monitoring to guide the treatment of OP in postmenopausal females undergoing TKA.

**Methods:**

Postmenopausal females with OP who underwent primary unilateral TKA were randomly divided into two groups (monitoring group and control group), given oral medication (alendronate, calcitriol, and calcium), and followed for 1 year. In the monitoring group, serum BTMs (C-telopeptide of type I collagen (CTX-I), N-terminal propeptide of type I procollagen (PINP), and 25(OH)D) were assessed preoperatively and repeated postoperatively; alendronate was withdrawn when CTX-I and PINP reached the reference interval; and calcitriol and calcium were withdrawn when 25(OH)D reached the reference interval. In the control group, oral medication was implemented for a uniform duration of 3 months. During the 1-year follow-up, the mean maximum total point motion (MTPM) of the tibial component, bone mineral density (BMD), visual analog scale (VAS) score, range of motion, and Oxford Knee Score (OKS) score were obtained.

**Results:**

In the monitoring group, BTM monitoring prolonged the medication duration, but did not cause more adverse reactions than in the control group. The mean MTPM values at 6 m and 12 m in the monitoring group were lower than those in the control group, and the BMD at 12 m in the monitoring group was significantly higher than that in the control group. Patients in the monitoring group had lower VAS scores at 6 m and higher OKS scores at 6 m and 12 m than those in the control group.

**Conclusion:**

In postmenopausal females with osteoporosis undergoing primary TKA, the application of BTM monitoring to guide the treatment of osteoporosis can enhance bone density, maintain prosthesis stability, and improve surgical outcome.

**Trial registration:**

ChiCTR ChiCTR-INR-17010495. Registered on 22 January 2017

## Background

Osteoarthritis (OA) is a common disorder that causes cartilage destruction in joints, particularly in knee joints. Total knee arthroplasty (TKA) is a successful procedure in treating severely damaged knee joints by providing pain relief and improving the mobility and stability of knees. Osteoarthritis and osteoporosis (OP) commonly coexist in postmenopausal females [1]. Due to estrogen deficiency, postmenopausal females have active bone turnover (bone resorption > bone formation) and increased bone loss, which can last for more than 10 years after menopause [2]. A more active bone turnover and decreased bone quality at the TKA bone-cement/implant interface in postmenopausal females brings potentially unstable fixation [3]. Aseptic loosening secondary to periprosthetic bone loss remains a major cause of TKA failure [4]. In addition, poor bone quality may negatively affect satisfaction with the surgical outcome of TKA [3]. For these reasons, preventing bone loss and prolonging the duration of a prosthesis is a vital issue in TKA [5].

Bisphosphonates are a class of first-line drugs used in the treatment of osteoporosis. They can reduce bone turnover, increase bone mass, and reduce fracture rate [6]. Bisphosphonates were shown to effectively reduce periprosthetic bone resorption and improve the long-term duration of knee prosthesis [7]. Guidance from the UK National Institute for Health and Care Excellence (NICE) recommends alendronate as the first choice of OP treatment [8], and oral administration is the most commonly used way for using bisphosphonates to treat osteoporosis [9]. It has been reported that oral alendronate not only prevented osteoclast-mediated bone loss and associated implant loosening in total hip arthroplasty (THA) [10], but also significantly reduced early postoperative periprosthetic bone loss in TKA patients [11]. However, the use of bisphosphonates to monitor the therapeutic effect in postmenopausal osteoporosis females undergoing TKA has not been clearly reported.

Bone turnover markers (BTMs) are biochemical markers in the blood and/or urine that are released during bone formation or bone resorption [12]. BTM can reflect systemic bone metabolism in a timely and accurate manner [13]. The examination of BTM is not expensive and noninvasive, and BTM can be measured repeatedly. Changes in BTMs after treatment might be more informative than changes in bone mineral density (BMD) measured by dual-energy X-ray absorptiometry (DEXA) [14]. BTMs may show large and rapid responses to the treatments used for osteoporosis, which may allow the best choice of dose and dose frequency [15]. The biochemical response to bisphosphonate therapy can be assessed using a decrease in BTMs to within a reference interval (RI) [15, 16].

BTMs are a useful adjunct for the therapeutic monitoring of osteoporosis, but no related research has reported on the use of bone turnover markers for monitoring osteoporosis treatment in postmenopausal females undergoing TKA. The purpose of this study was to investigate the clinical value of perioperative BTM monitoring to guide the treatment of osteoporosis in postmenopausal females after TKA. The hypothesis was that BTM monitoring in postmenopausal females with OP can bring potentially stable fixation and achieve satisfactory surgical outcomes after TKA.

## Methods

### Patients

**Fig. 1:**
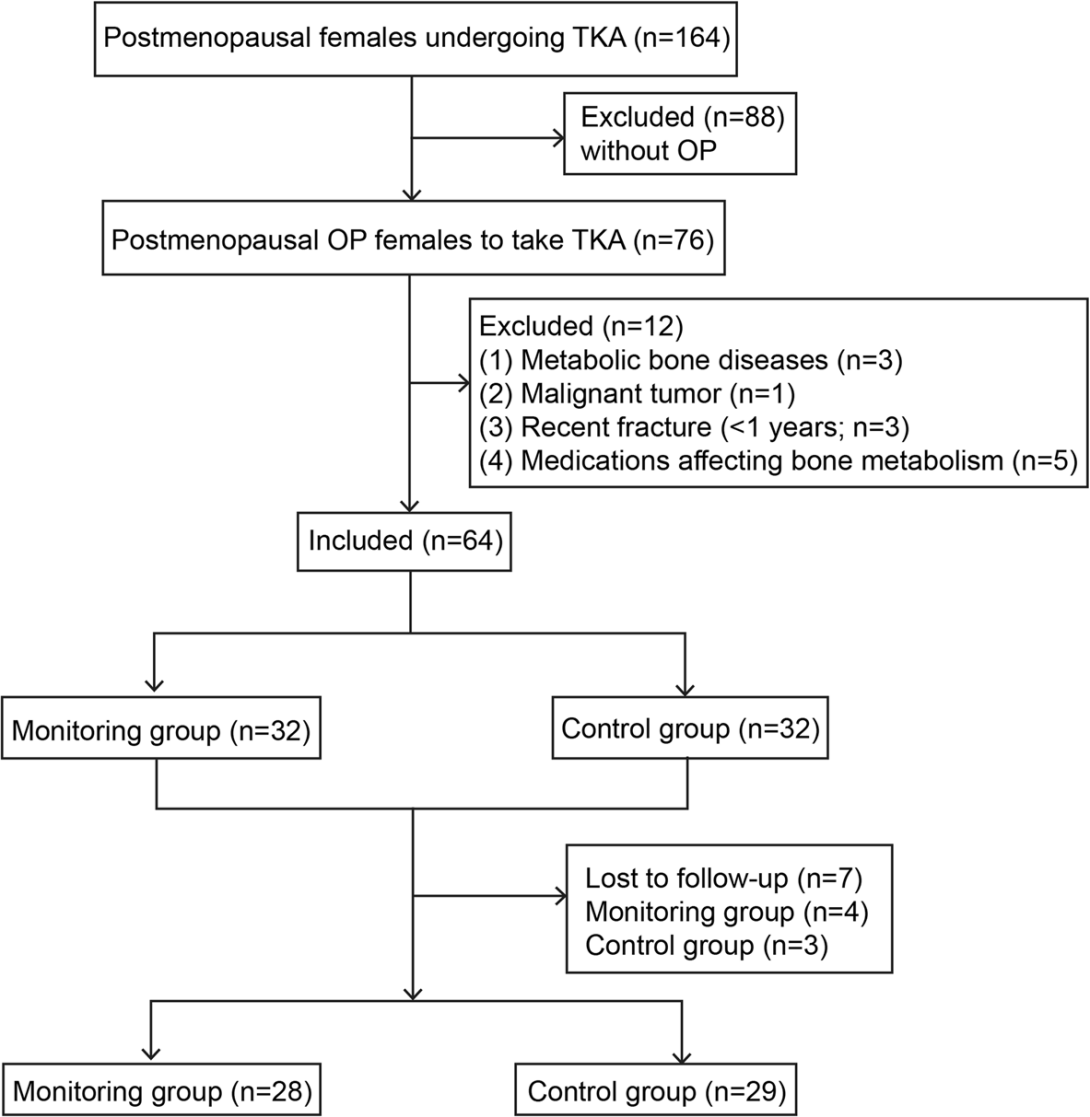
Patient flow diagram

This was a randomized controlled intervention trial with consecutive enrollment. From April 2017 to December 2018, female OA patients (aged 55–75 years) at the Second Affiliated Hospital of Xi'an Jiaotong University who underwent primary TKA were screened. The females in the postmenopausal period (at least 1 year of amenorrhea), with lumbar/hip BMD expressed as *T* score < 2.5 standard deviation (SD) [17], were included. The exclusion criteria were as follows: (1) other metabolic bone diseases; (2) malignant tumor; (3) fracture within 1 year; and (4) history of taking vitamin D, calcitonin, estrogen, or bisphosphonates within 1 year. This study was registered by the Chinese Clinical Trial Registry (ChiCTR-INR-17010495) and approved by the Medical Ethics Committee of the Second Affiliated Hospital of Xi’an Jiaotong University (2017011). All participants gave written informed consent and agreed for the publication. Finally, 64 patients were included (Fig. 1).

### Surgery

All operations were performed under general anesthesia by Professor Kunzheng Wang. Total knee arthroplasty was performed using cemented, posterior-stabilized prostheses through an anterior median incision, and patella arthroplasty was performed. After the operation, all patients underwent active and passive knee exercises of full range of motion and began walking with crutches or walking aids when active knee flexion reached 90°. The age, body weight index (BMI, kg/m^2^), years since menopause, operation time, and ambulation time of all patients were recorded. All included patients experienced a 1-year follow-up, and 7 patients were lost to follow-up (Fig. 1).

### Grouping

All patients were randomized into two groups (monitoring group and control group). After the operation, all patients were given oral anti-osteoporosis treatment, which was alendronate (70 mg/week; Fosamax, Merck, Germany) + calcitriol (0.50 μg/day; Adcal D3, ProStrakan, UK) + calcium (Ca) carbonate (0.3 g/day; D-Cai, Ace, UK). In the monitoring group (*n* = 28), serum BTM (CTX-I, P1NP) and 25(OH)D values were assessed preoperatively (baseline) and repeated postoperatively at 1, 3, 5, 7, 9, and 12 months; alendronate was withdrawn when CTX-I and PINP reached the reference interval, and calcitriol and calcium were withdrawn when 25(OH)D reached the reference interval. The reference intervals in postmenopausal females were CTX-I < 1.008 ng/mL and PINP < 95 ng/mL [18]. In the control group, oral medication was implemented uniformly for 3 months, and no BTM values were assessed.

### Assessment of bone turnover markers

The International Osteoporosis Foundation (IOF) and International Federation of Clinical Chemistry recommend C-telopeptide of type I collagen (CTX-I) as a serum bone resorption marker and N-terminal propeptide of type I procollagen (PINP) as a serum bone formation marker [14]. Serum CTX-I and PINP were assayed using an electrochemiluminescence immunoassay (Elecsys 1010 Analytics, Roche Diagnostics, Germany). Serum 25(OH)D was measured by chemiluminescence assay (Architect 1000, Abbott, USA). Fasting blood samples of patients were collected between 7 a.m. and 8 a.m. before the operation (baseline).

### Tibial component fixation

Radiostereometric analysis (RSA) is a precise and highly accurate tool for the assessment of tibial component migration after TKA [19–21]. RSA was performed at 6 and 12 months postoperatively. An RSA parameter, the mean maximum total point motion (MTPM) was used to analyze the stability of the tibial component fixation. MTPM is defined as the largest three-dimensional translation of the tibial component [22]. The detailed RSA procedure referred to a previous study [21].

### Bone mineral density

At 1 to 3 days pre-operation (baseline) and 12 months postoperatively, BMD (g/cm^2^) values of the lumbar spine or proximal femur were measured by DEXA using a densitometer (Discovery A; Hologic Inc., Bedford, USA). The *T* scores were calculated separately using the following formula: (measured BMD-young adult mean BMD)/young adult population SD [23]. A *T* score < − 2.5 was classified as osteoporosis [17].

### Knee pain and function scores

At 1 to 3 days preoperatively (baseline) and 3, 6, and 12 months postoperatively, visual analog scale (VAS) [24], range of motion (ROM), and Oxford Knee Score (OKS) [25] were recorded for all patients. The range of motion was the active ROM of the knees. In the OKS, each question was scored from 1 to 5, and responses were then totaled to obtain a total score between 12 and 60 [25].

### Statistical analysis

We calculated the sample size at a website (url: http://powerandsamplesize.com/Calculators/). We selected the power (1-β) as 0.80 and type I error rate (α) as 5%. The calculated sample size was 63.

All continuous variables are expressed as the mean ± SD. All data were managed through SPSS (IBM SPSS Statistics 19, USA). All continuous variables were analyzed by the normality test. When the variables were normally distributed, the independent *t* test was used; when the variables were not normally distributed, the Mann-Whitney *U* test was used. The *χ*^2^ test was used to compare the categorical variables. The level of significance was < 0.05 for all tests.

## Results

### Demographic results

A total of 64 patients were included, with 32 patients in each group. During the follow-up period, 4 patients in the monitoring group and 3 patients in the control group were lost to follow-up. Finally, there were data available for 28 patients in the monitoring group and 29 patients in the control group. The average age of the patients was 65.1 ± 6.0 years, the average BMI was 23.4 ± 4.6 kg/m^2^, and the average years since menopause was 13.4 ± 6.8 years. The age, BMI, years since operation time, and ambulation time in the two groups exhibited no significant differences (*p* < 0.05; Table 1).

**Table 1:** Characteristics of the patients in each group

	Monitoring group (*n* = 28)	Control group (*n* = 29)	Total	*p* value
Age (years)	65.6 ± 6.2	64.6 ± 5.8	65.1 ± 6.0	0.538
BMI (kg/m^2^)	22.9 ± 4.7	23.9 ± 4.6	23.4 ± 4.6	0.448
Years since menopause	14.9 ± 7.1	11.9 ± 6.4	13.4 ± 6.8	0.107
Operation time (min)	77.0 ± 8.8	79.7 ± 8.3	78.4 ± 8.6	0.245
Ambulation time (days)	3.3 ± 1.5	2.9 ±1.0	3.1 ± 1.3	0.209

*BMI* body weight index

### Medication duration

The alendronate duration, calcitriol + Ca duration, and total medication duration in the monitoring group were significantly higher than those in the control group (*p* < 0.05), but the adverse reaction rates between the two groups were not significantly different (*p* > 0.05). In addition, all the adverse reactions were mild and the patients could tolerate the adverse reactions (Table 2).

**Table 2:** Medication duration and adverse reactions of treatment in the BTM monitoring group and the control group

	Monitoring group (*n* = 28)	Control group (*n* = 29)	*p* value
Alendronate duration (days)	236 ± 71	90 ± 0	0.000
Calcitriol + Ca duration (days)	214 ± 72	90 ± 0	0.000
Total medication duration (days)	259 ± 72	90 ± 0	0.000
Adverse reaction	5 (17.9%)^a^	2 (6.9%)^b^	0.392

*Ca* calcium

^a^Three patients had gastrointestinal reaction and two patients had muscular soreness

^b^One patient had gastrointestinal reaction and one patient had headache

### Changes in BTMs in the monitoring group

**Fig. 2:**
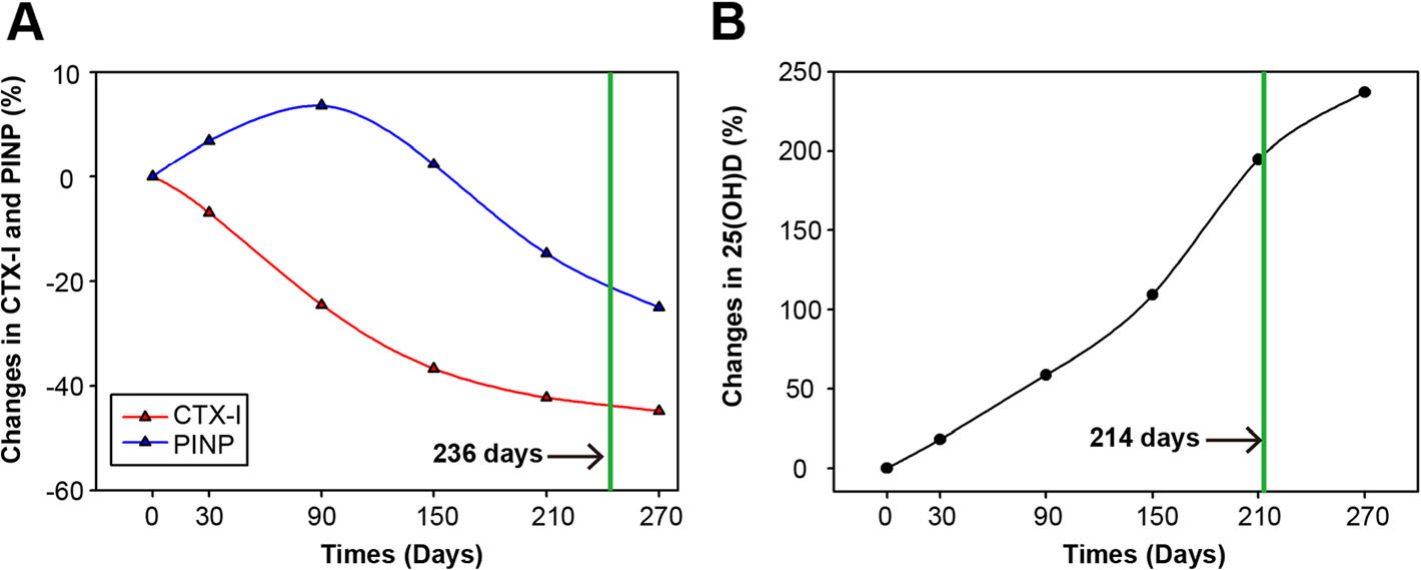
Serum levels of CTX-I and PINP (**a**) and 25(OH)D (**b**) in the monitoring group. The green line is the drug withdrawal time (**a** alendronate, **b** calcitriol + Ca)

The CTX-I level in the monitoring group gradually decreased after treatment and reached a plateau at approximately 150 days (Fig. 2a) However, the PINP level increased slightly before approximately 80 days and then gradually decreased until drug withdrawal at an average of 236 days (Fig. 2a). The level of 25(OH)D showed a trend of continuous increase after treatment until drug withdrawal at an average of 214 days (Fig. 2b).

### Tibial component fixation and bone mineral density

At 6 and 12 months, the mean MTPM values in the monitoring groups were all significantly higher than those in the control group (*p* < 0.05). The BMD values before the operation in the two groups showed no significant difference (0.58 ± 0.06 vs. 0.59 ± 0.05; *p* > 0.05). At 12 m after the operation, the BMD value of the monitoring group increased by 9.7% compared to that before the operation, while the BMD value of the control group decreased by 3.2% compared to that before the operation. The changes in BMD at 12 m between the two groups exhibited an obviously statistical difference (*p* < 0.05) (Table 3).

**Table 3:** Comparison of mean MTPM and BMD during the 1-year follow-up

	Monitoring group (*n* = 28)	Control group (*n* = 29)	*p* value
Mean MTPM at 6 m (mm)	0.17 ± 0.07	0.22 ± 0.08	0.011
Mean MTPM at 12 m (mm)	0.42 ± 0.15	0.57 ± 0.20	0.003
BMD at baseline (g/cm^2^)	0.58 ± 0.06	0.59 ± 0.05	0.509
Changes in BMD at 12 m (%)	9.7 ± 16.6	− 3.2 ± 13.3	0.002

*MTPM* maximum total point motion, *BMD* bone mineral density

### Knee pain and function scores

There were no significant differences in VAS scores and ROM or OKS scores between the two groups before the operation (*p* > 0.05). The VAS score at 6 m in the monitoring group were significantly lower than those in the control group (*p* < 0.05), and the OKS scores at 6 and 12 months in the monitoring group were significantly higher than those in the control group (*p* < 0.05). There was no significant difference in the ROM values between the two groups at any time point (*p* > 0.05) (Table 4).

**Table 4:** Comparison of knee pain and function scores during the 1-year follow-up

Knee parameters	Groups	Baseline	3 m	6 m	12 m
VAS score	Monitoring group	6.6 ± 1.9	3.6 ± 1.6	2.0 ± 1.3	1.8 ± 1.3
	Control group	6.6 ± 1.6	3.5 ± 1.6	2.8 ± 1.1	1.8 ± 1.1
	*p* value	0.906	0.292	0.042	0.811
ROM (°)	Monitoring group	101.8± 15.6	116.4 ± 11.7	113.8 ± 11.6	108.0 ± 14.4
	Control group	103.3 ± 18.4	110.4 ± 14	110.5 ± 7.5	110.7 ± 9.6
	*p* value	0.743	0.099	0.219	0.418
OKS score	Monitoring group	46.5 ± 7.5	25.2 ± 5.5	22.4 ± 6.0	21.1 ± 4.1
	Control group	48.1 ± 7.0	26.4 ± 5.8	19.0 ± 3.9	18.4 ± 3.9
	*p* value	0.419	0.425	0.014	0.017

*VAS* visual analog scale, *ROM* range of motion, *OKS* Oxford Knee Score

## Discussion

Most postmenopausal females with osteoporosis appear to have some form of osteoarthritic impairment [26]. Postmenopausal bone loss is believed to occur as a result of an increase in the rate of bone turnover and a negative imbalance between bone formation and resorption [27], which may affect the surgical outcome of TKA and increase the incidence of prosthesis loosening [3]. Bone turnover markers provide timely bone turnover information that allows for earlier intervention in osteoporosis care [13]. As access to DXA scans is becoming more limited because of cost and insurance restrictions, BTM may become increasingly used in the therapeutic monitoring of osteoporosis [28]. To analyze the impact of BTM monitoring on improving osteoporosis treatment outcomes, prosthesis stability, and TKA surgical outcome, BTM monitoring was applied to guide osteoporosis treatment and compared with empirical treatment in postmenopausal osteoporosis females undergoing TKA.

In this study, the decrease in values of bone resorption marker (CTX-I) occurred rapidly after starting treatment with antiresorptive agents (alendronate). In contrast, the change in BMD occurs over months or years [15], so that BTM may provide earlier information on the response to anti-osteoporosis treatment than BMD. We observed a decrease in the bone resorption marker (CTX-I) earlier than in the bone formation marker (PINP) after treatment of postmenopausal osteoporosis, which has been reported by other studies [15, 29]. After 210 days of anti-osteoporosis treatment, CTX-I decreased by approximately 43% and PINP decreased by approximately 16%, indicating that bone resorption markers decreased by a greater magnitude than bone formation markers in response to bisphosphonate treatment. This phenomenon is caused by the pharmacological mechanism of alendronate, because alendronate directly inhibits bone resorption by osteoclasts and accordingly results in relatively rapid decreases in bone resorption markers; this causes a decrease in bone formation markers due to physiologic mechanisms linking osteoclast and osteoblast activity [13].

BTM can quickly and accurately reflect the state of bone resorption and bone formation, providing guidance and assistance when using bisphosphonate treatment. In this study, the treatment duration of the monitoring group was significantly extended, but the adverse reactions did not significantly increase. These results indicated that treatment under the monitoring of BTM was primarily safe. We measured BMD before and 1 year after TKA and found that the BMD values in the monitoring groups increased by 9.7%, while the BMD values in the control group decreased by 3.2%. This meant that the duration of the empirical treatment (3 months) was not long enough to achieve a long-term increase in BMD. The BMD reduction in the control group was probably attributed to the reduced mobility during rehabilitation [30]. BTM monitoring pays close attention to bone turnover in osteoporosis patients. When bone resorption and bone formation markers were within the reference intervals, bone turnover tended to be relatively balanced. Early decreases in bone turnover markers at 6–12 months of bisphosphonate treatment correlate with a long-term increase in bone mineral density [31].

Bone metabolism and bone quality are important for the initial stability of prostheses [3, 32, 33]. Bone loss in osteoporosis patients may have a negative effect on TKA, such as reduced stability, reduced lifetime of the prosthesis, and an increased rate of revision [3]. Reasons for periprosthetic bone loss include initial implant micromotion and migration [7]. This study placed emphasis on the initial migration of the prosthesis and used the mean MTPM of the tibial component to analyze prosthesis stability. Oral bisphosphonate significantly reduced early postoperative periprosthetic bone loss [11] and prevented osteolysis and aseptic loosening in TKA patients [7]. We found that the mean MTPM values at 6 and 12 months in the monitoring groups were all significantly higher than those in the control group. Under the monitoring of BTMs, patients might experience a more balanced bone turnover, a gradual increase in bone density, and a higher degree of prosthesis stability compared to the control.

Approximately one in five patients may be dissatisfied with their elective TKA, with dissatisfaction mainly focused on pain relief and function recovery [34]. The decrease in BMD in postmenopausal females with osteoporosis may affect the surgical results of TKA [3], particularly without effective and reasonable anti-osteoporosis treatment. Bisphosphonates improved the quality of life scores of postmenopausal females undergoing cementless THA [35]. In this study, we used the VAS score and ROM and OKS score to evaluate patient satisfaction with the surgical outcome, and the OKS score was mainly used to evaluate knee pain and daily function. We found that the VAS score at 6 months and OKS scores at 6 and 12 months after surgery in the BTM monitoring group were higher than those in the control group, indicating a higher satisfaction with the surgical outcome in the monitoring group. These results were likely because of the pain-reducing and stability-maintaining effects of alendronate treatment.

Although BTM monitoring prolonged the treatment duration of postmenopausal osteoporosis females undergoing TKA, it was still an effective way to guide osteoporosis treatment. Compared to empirical treatment, BTM monitoring treatment showed a valuable effect on improving the treatment outcomes of osteoporosis, thereby sustaining initial prosthesis stability. With lower pain scores and higher function scores, BTM monitoring treatment also achieved a satisfactory surgical outcome.

There were several limitations to this study. Firstly, the sample size was relatively small due to the specific requirements of patient recruitment in this study. Some patients refused to collect blood samples after operation. Second, the follow-up period (1 year) in this study was relatively short. The treatment of osteoporosis is a long-term procedure, and the bone mineral density is in a dynamic change after osteoporosis treatment. A prolonged follow-up period may supply more information after treatment and monitoring. Third, the sample may have selection bias. The female OA patients aged 55–75 years were selected, but increase of age (> 65 years) may also affect the BMD except for menopause. Our results need to be confirmed in larger sample sizes and with a longer follow-up period in the future.

## Conclusion

The application of BTM monitoring to guide the treatment of osteoporosis in postmenopausal females can significantly improve bone density, prosthesis stability, and surgical outcomes after TKA, which may be of great significance in reducing the incidence of prosthesis loosening and improving surgical satisfaction in postmenopausal females with osteoporosis undergoing TKA.

## Data Availability

Data sharing not applicable to this article as no datasets were generated or analyzed during the current study.

## Abbreviations

OP: Osteoporosis
OA: Osteoarthritis
TKA: Total knee arthroplasty
BTM: Bone turnover marker
CTX-I: C-telopeptide of type I collagen
PINP: N-terminal propeptide of type I procollagen
MTPM: Maximum total point motion
BMD: Bone mineral density
NICE: National Institute for Health and Care Excellence
THA: Total hip arthroplasty
DEXA: Dual energy X-ray absorptiometry
RI: Reference interval
SD: Standard deviation
BMI: Body weight index
Ca: Calcium
IOF: International Osteoporosis Foundation
RSA: Radiostereometric analysis
VAS: Visual analog scale
ROM: Range of motion
OKS: Oxford Knee Score
